# Silver Jubilee of Stroke Thrombolysis With Alteplase: Evolution of the Therapeutic Window

**DOI:** 10.3389/fneur.2021.593887

**Published:** 2021-03-01

**Authors:** Yuanmei Pan, Guowen Shi

**Affiliations:** Ren Ji Hospital, School of Medicine, Shanghai Jiao Tong University, Shanghai, China

**Keywords:** stroke, thrombolysis (tPA), alteplase, acute cerebral ischemic stroke, neurocritical care

## Abstract

In 1995, the results of a landmark clinical trial by National Institute of Neurological Disorders and Stroke (NINDS) made a paradigm shift in managing acute cerebral ischemic stroke (AIS) patients at critical care centers. The study demonstrated the efficacy of tissue-type plasminogen activator (tPA), alteplase in improving neurological and functional outcome in AIS patients when administered within 3 h of stroke onset. After about 12 years of efforts and the results of the ECASS-III trial, it was possible to expand the therapeutic window to 4.5 h, which still represents a major logistic issue, depriving many AIS patients from the benefits of tPA therapy. Constant efforts in this regards are directed toward either speeding up the patient recruitment for tPA therapy or expanding the current tPA window. Efficient protocols to reduce the door-to-needle time and advanced technologies like telestroke services and mobile stroke units are being deployed for early management of AIS patients. Studies have demonstrated benefit of thrombolysis guided by perfusion imaging in AIS patients at up to 9 h of stroke onset, signifying “tissue window.” Several promising pharmacological and non-pharmacological approaches are being explored to mitigate the adverse effects of delayed tPA therapy, thus hoping to further expand the current tPA therapeutic window without compromising safety. With accumulation of scientific data, stroke organizations across the world are amending/updating the clinical recommendations of tPA, the only US-FDA approved drug for managing AIS patients. Alteplase has been a part of our neurocritical care and we intend to celebrate its silver jubilee by dedicating this review article discussing its journey so far and possible future evolution.

## Introduction

Acute cerebral stroke is a cerebrovascular disease characterized by an acute compromise of cerebral perfusion or vasculature. It is fundamentally divided into acute intracerebral hemorrhagic stroke (AHS) and acute cerebral ischemic stroke (AIS), with the latter constituting for about 85% of the total cases (1). Before 1995, both AHS and AIS patients reaching critical care centers were treated and cared alike, with no efforts made to differentiate between the two with respect to their management. However, in 1995, a landmark clinical trial by National Institute of Neurological Disorders and Stroke (NINDS) in managing AIS with Alteplase, a recombinant tissue-type plasminogen activator (tPA), was published (2). The study initiated a paradigm shift in managing AIS patients, thus triggering the need to quickly differentiate between AHS and AIS conditions. However, the trial suggested a narrow treatment window, 3 h of stroke onset, a major logistic issue that remains challenging even today. Treatment beyond this window was shown to be detrimental to patients' health than being of any help. Efforts have been since made to expand this treatment window to also include AIS patients reaching emergency care beyond the 3 h window under the benefits of tPA therapy. After over 12 years of constant efforts, European Cooperative Acute Stroke III (ECASS III) study was able to expand this treatment window to 4.5 h (3). About 8 years later, a meta-analysis of five mechanical thrombectomy trials showed that by bridging tPA with intra-arterial thrombectomy, the treatment window can be expanded to 6 h (4). In 2018, we learnt that a subset of AIS patients with large vessel occlusion were eligible for intra-arterial thrombectomy at up to 24 h of stroke onset (5). Recent studies are suggesting of tissue-window and not the time-window to guide tPA treatment in AIS patients. Further, few potential pharmacological molecules are being studied as conjunctive to tPA therapy to minimize its post-window adverse effects and, thus, possibly increase the therapeutic window. Hence, at the silver jubilee of tPA in managing AIS patients, we are prompted to look into the 25 years of evolution of the tPA therapeutic window, and at its promising future directions, all of which are summarized below.

## Tissue-Type Plasminogen Activator

**Figure 1:**
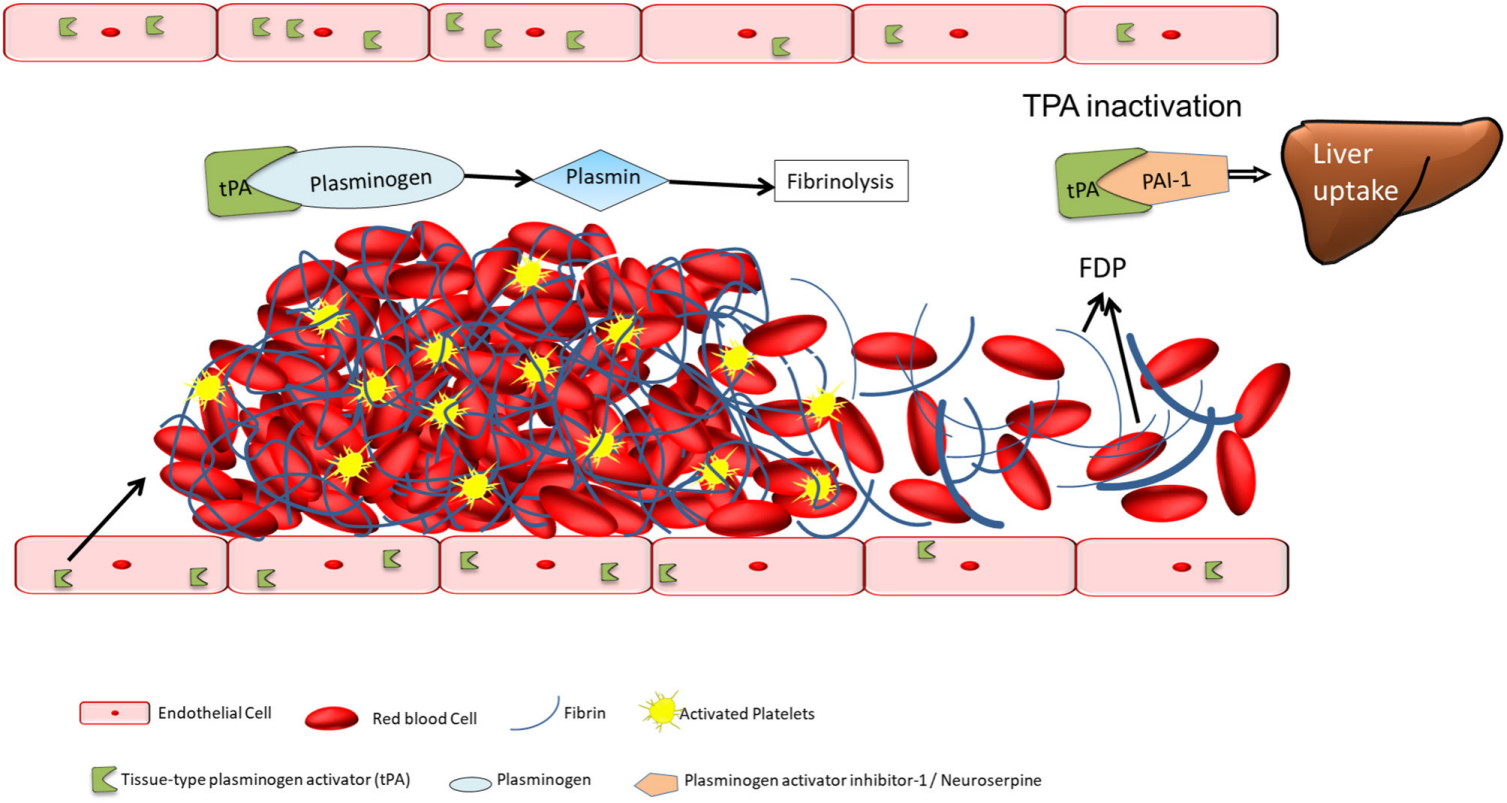
The catalystic action of tissue-type plasminogen activator in thrombolysis.

Tissue-type plasminogen activator (tPA) is a serine protease enzyme that functions as an essential catalyst in thrombolysis (Figure 1). Found in endothelial cells, tPA is released in response to aggregation of activated platelets (6) and catalyzes the conversion of plasminogen to plasmin, by cleaving the zymogen plasminogen at its Arg561-Val562 peptide bond, thus aiding in thrombolysis (7). Its activity is eventually terminated by binding with plasminogen activator inhibitor 1, or neuroserpin—a neuronal specific inhibitor of tPA, forming an inactive complex, which is removed from the circulation by LDL-receptor-related protein 1 in liver (7). Synthetic version of tPA (ex., Alteplase) can be made in labs through recombinant biotechnology, some of which are modified to amplify their pharmacokinetic and pharmacodynamic properties (ex., Reteplase and Tenecteplase). Alteplase, the synthetic version of normal human tPA, is FDA approved for treating AIS, myocardial infarction with ST-elevation, acute pulmonary embolism and with central venous access devices (8). The modified tPA reteplase and tenecteplase possess longer half-life, translating into longer duration of action, and are indicated for management of acute pulmonary embolism and acute myocardial infarction, respectively (9, 10).

Post-stroke administration of tPA is associated with its own risks, the most devastating of which is hemorrhagic transformation (HT) by thrombolytic and non-thrombolytic actions (11, 12). Thrombolytic activity of tPA facilitates reperfusion, and post-ischemic reperfusion has been shown to degrade basal lamina and disrupt blood brain barrier (BBB) in as early as 10 min (13). Post stroke, in humans, the extent of BBB disruption and its permeability may alter in the first 24 h, resulting in an early HT which is different from a late HT that is witnessed after 24 h, where a persistent disruption is witnessed that lasts for weeks (14). Reactive oxygen species (ROS), blood-derived matrix metalloproteinase (MMP)-9 and brain-derived MMP-2 contribute to early HT, while brain-derived MMP-3, MMP-9, and other proteases guide late HT along with active vascular remodeling and neuroinflammation (12). The tPA induced non-thrombolytic mode of HT is facilitated through increasing the activity of MMPs (MMP-2, MMP-3, MMP-9) through protease activated receptor-1, lipoprotein receptor protein receptor signaling and degranulation of neutrophils, and of PDGF-CC through PDGF-receptor-α signaling (12).

## Twenty 5 Years of Evolution of tPA Therapeutic Window

**Figure 2:**
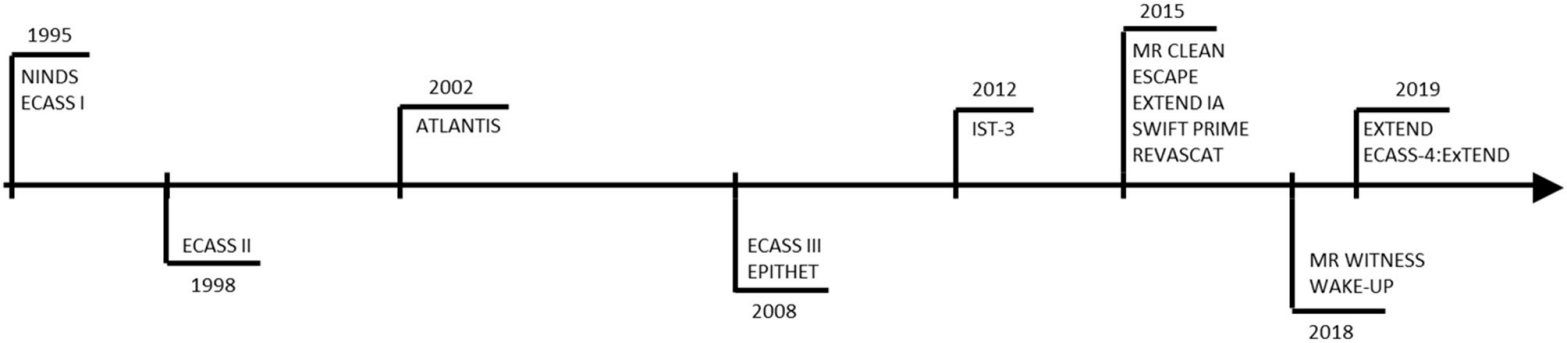
Clinical trials representing 25 years of evolution of therapeutic window of Alteplase.

Over the past 25 years several clinical trials of international importance has been, and are being, conducted (Figure 2 and Table 1). It all started 25 years ago with two interlinked clinical trials by National Institute of Neurological Disorders and Stroke (NINDS), the results of which were reported together (2). Based on the results from two previous dose escalation studies (15), three therapeutic windows, 0–90, 91–180, and 0–180 min from stroke onset, for tPA administration was assessed in NINDS trials. The neurological assessments at 24 h of tPA (0.9 mg/kg) treatment, addressed in the first part of the study, showed no significant improvement compared to placebo. However, the second part of the study demonstrated a long term clinical benefit of tPA treatment in AIS patients, treated within 3 h of stroke onset, as measured by “global odds ratio for a favorable outcome.” The study also reported a significant increase in symptomatic intracerebral hemorrhage (sICH) within 36 h of tPA treatment (6.4 vs. 0.6%; *p* < 0.001). The severity of baseline neurological deficit and brain edema were identified as the variables associated with this increased risk of sICH, which was reported later (16). A few concerns were raised over choosing National Institute of Health Stroke Scale (NIHSS) and global composite scale over other more efficient scales and the imbalance in the baseline stroke severity that might potentially favor the tPA group. This resulted in a subsequent independent reanalysis of the NINDS trial data, which refuted all the concerns, thus supporting the use of tPA to treat AIS patients within 3 h of stroke onset (17).

**Table 1:** The results and implications of the key trials of intravenous thrombolysis for ischemic stroke.

**Study**	**Hypothesis**	**TPA Time Window**	**Sample size**	**Main outcome measure**	**Results**	**Implications**
National Institute of Neurological Disorders and Stroke (NINDS)	Part 1: AIS patients receiving tPA would demonstrate early (24 h) clinical improvement, compared to placebo group Part 2: AIS patients receiving tPA would recover with minimal or no deficit at 3 months after treatment, compared to placebo group	0–3 h	Part 1: 291 Part 2: 333	Part 1: ≥4 points over baseline NIHSS score Part 2: BI MRS GOS NIHSS	Part 1: No significant difference in neurologic improvement at 24 h between the tPA and placebo group Part 2: Patients treated with tPA we 30% more likely to have no or minimal disability at 3 months after treatment, compared to placebo group ICH was significantly higher in tPA group (*p* < 0.001) Mortality at 3 months was little lower in tPA group compared to placebo, although not significant	Treatment with tPA within 3 h of stroke onset improved the clinical outcome at 3 months in AIS patients, despite an initial increase in ICH incidence
ATLANTIS	To test the efficacy and safety of rt-PA in patients with acute ischemic stroke when administered between 0 and 5 h after symptom onset	A: 0–3 h B: 3–5 h	A: 61 B: 613	A/B: At 90 days NIHSS≤1 BI≥95 MRS*≤* 1 GOS=1	A: Very favorable outcome at 90 days B: No significant benefit with increased ICH	Recommends tPA administration in <3 h of onset of stroke symptoms, while it doesn't support tPA administration beyond 3 h window
ECASS III	The efficacy of alteplase administered in patients with acute ischemic stroke can be safely extended to a time window of 3 to 4.5 h after the onset of stroke symptoms	3–4.5 h	821	MRS≤1 Composite analysis of: BI, MRS, GOS, NIHSS	Both primary and secondary outcome was significantly more in tPA group compared to placebo group	As compared with placebo, intravenous alteplase administered between 3 and 4.5 h after the onset of symptoms significantly improved clinical outcomes in patients with acute ischemic stroke
EPITHET	Primary: Greater attenuation of infarct growth in patients with an imaging mismatch who received alteplase than in those who received placebo Secondary: Reperfusion, good neurological outcome, and good functional outcome would be more likely in mismatch patients who received alteplase than in those who received placebo, Incidence of symptomatic ICH would be associated with larger baseline DWI volumes in patients who received alteplase	3–6 h	100	Primary: infarct growth between baseline DWI and the day 90 T2 lesion in mismatch patients. Secondary: reperfusion, good neurological outcome, and good functional outcome	Reduced infarct growth in alteplase group, although not significant. Reperfusion was common with better neurological outcome and functional outcome in alteplase group	Alteplase was not significantly associated with infarct growth, but significantly associated with increased reperfusion in patients who had mismatch
IST-3	0·9 mg/kg rt-PA (maximum 90 mg) given to adult patients of all ages with acute ischaemic stroke, within 6 h of symptom onset, increased the proportion of people who were alive and independent at 6 months	0–6 h	3,035	OHS ≤ 2 at 6 months	A similar number of patients were alive and independent in both the tPA and control group at 6 months, with a significant shift in OHS scores toward tPA group	Despite the early hazards, alteplase administration in <6 h of onset of stroke symptoms improved the functional outcome, and this benefit did not diminish in elderly patients (>80 years of age)
MR WITNESS	A quantitative diffusion-FLAIR mismatch (qDFM) can be used in place of time from last known well to identify stroke patients with unwitnessed symptom onset who can safely be treated with thrombolytic therapy.	4.5–24 h since last well-known	80	Primary: Risk of symptomatic intracranial hemorrhage (sICH) with pre-planned stopping rules. Secondary: symptomatic brain edema risk, and functional outcomes of 90-day MRS	SICH: 1 case Symptomatic edema: 3 cases MRS≤1 in 39% cases	Intravenous thrombolysis within 4.5 h of symptom discovery in patients with unwitnessed stroke selected by qDFM, who are beyond the recommended time windows, is safe.
WAKE UP	Patients with stroke with an unknown time of onset and features suggesting recent cerebral infarction on magnetic resonance imaging (MRI) would benefit from thrombolysis with the use of intravenous alteplase	Unknown time of onset [<4.5 h as indicated by ischemic lesion that was visible on MRI diffusion-weighted imaging but no parenchymal hyperintensity on FLAIR]	503	Primary: MRS≤1 Secondary: Lower ordinal score on MRS in alteplase grouip than placebo group	A significantly higher number of patients in alteplase group demostrated favorable outcome at 90 days	In patients with acute stroke with an unknown time of onset, intravenous alteplase guided by a mismatch between diffusion-weighted imaging and FLAIR in the region of ischemia resulted in a significantly better functional outcome and numerically more intracranial hemorrhages than placebo at 90 days
EXTEND	Intravenous thrombolysis with alteplase initiated between 4.5 and 9.0 h after stroke onset or on awakening with stroke symptoms (for which the time of onset was not known) would provide a benefit in patients who had a small core volume of cerebral infarction that was disproportionate to a larger area of hypoperfusion	4.5–9 h after the onset of stroke or on awakening with stroke	225	MRS≤1	A significantly higher number of patients in alteplase group demostrated favorable outcome at 90 days	In AIS patients with salvageable brain tissue, alteplase treatment within the specified time results in higher percentage of patients with no or minor neurologic symptoms than palcebo
ECASS-4-EXTEND	ischemic stroke patients selected with significant penumbral mismatch on magnetic resonance imaging (MRI) at 4.5–9 h after onset of stroke will have improved clinical outcomes when given intravenous rt-PA (alteplase) compared to placebo	4.5–9 h after the onset of stroke or on awakening with stroke	111	MRS≤1	Patients with mismatch showed odds ratio in favor of treatment benefit of alteplase at 90 days	A DWI-FLAIR mismatch in the region of ischemia as imaging based surrogate parameter for patient selection for i.v. rt-PA should be strongly pursued

**Figure 3:**
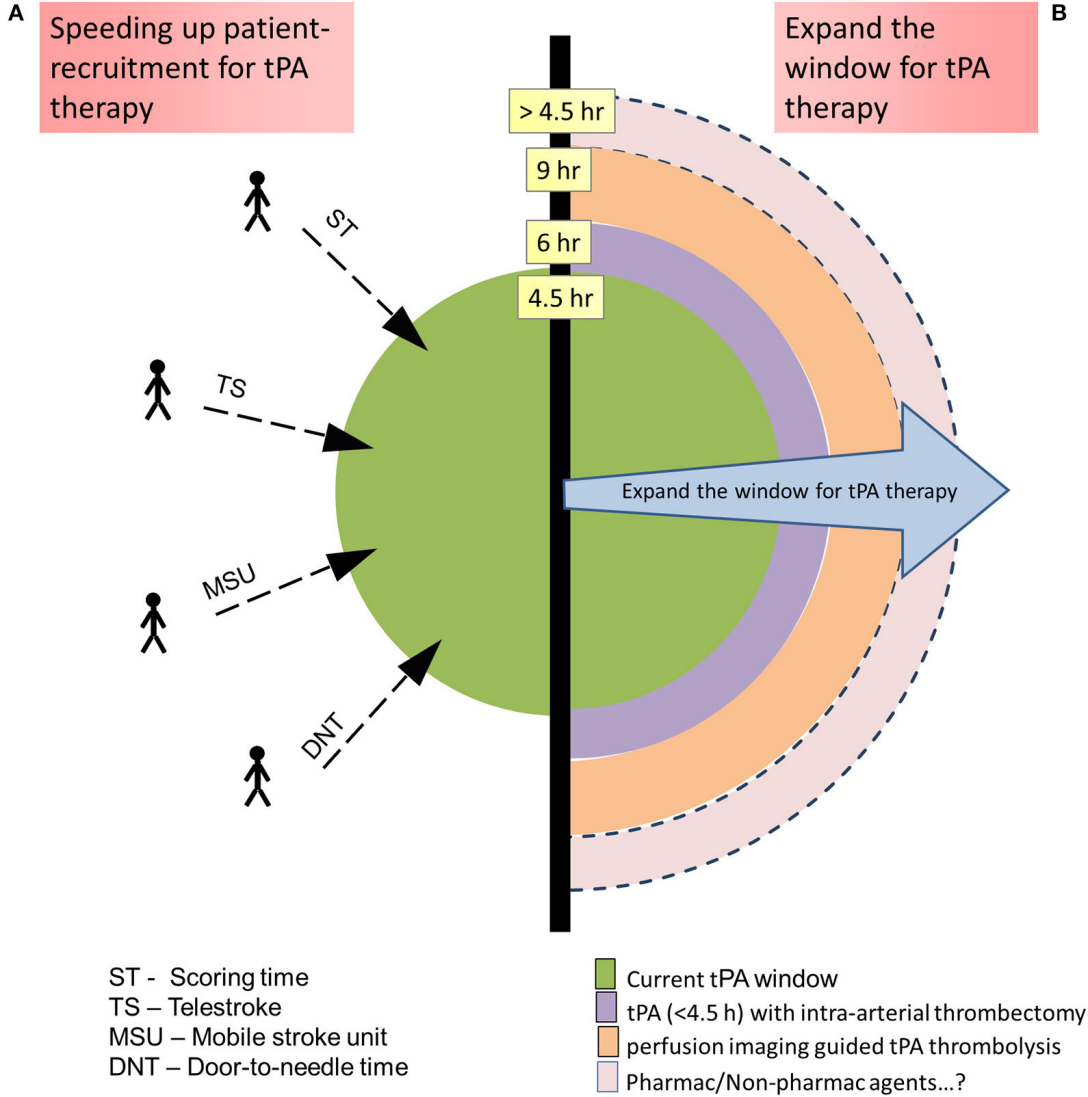
Methods to increase the number of acute ischemic stroke patients' eligibility for tissue-type plasminogen activator (tPA) therapy: **(A)** By speeding up the patient recruitment so that they fall within the recommended tPA therapeutic window. **(B)** Expanding the tPA therapeutic window with added technologies/therapies.

Within months after publishing of NINDS results, the European Union also published the results of ECASS I study where a higher dose of tPA (1.1 mg/kg) was administered to AIS patients within 0–6 h of stroke onset (18). The studied primary outcome measures failed to elicit significant difference between tPA and placebo group, which was attributed to significant number of protocol violations (~17%) included in the analysis. Further a significant increase in parenchymal hemorrhages and mortality was reported in tPA treated group compared to placebo-treated patients. Soon, ECASS II was designed and executed with a lower tPA dose (0.9 mg/Kg) administered to AIS patients at up to 6 h of stroke onset (19). However, with no significant difference between the assessed primary end point for treatment and placebo group (*p* = 0.277), it was concluded that tPA was not beneficial in AIS patients when administered under the study parameters. Another contemporary study was “the alteplase thrombolysis for acute non-interventional therapy in ischemic stroke” (ATLANTIS) study which was initially designed to assess the safety and efficacy of tPA administration in AIS patients within 0–6 h therapeutic window, and was referred to as part-A (20) (Figure 3). The results found no significant benefits of alteplase administered between 0 and 6 h after stroke onset, particularly those treated after 3 h. Further, AIS patients receiving tPA at 5–6 h of stroke onset demonstrated a significant increase in sICH. Due to these safety concerns the window was changed to 0–5 h and continued as ATLANTIS part-B, the results of which was reported according to two subsets of therapeutic window; 0–3 and 3–5 h (21, 22). The patients in 3–5 h window of tPA treatment showed no better recovery and functional outcome compared to placebo group, added by increased sICH and fatal ICH (*p* < 0.001). However, the results of patients within the 3 h window of tPA treatment was comparable to NINDS trail results, where they demonstrated a better functional outcome at 90 days, compared to placebo. Hence, the ATLANTIS study recommended the 3 h therapeutic window for tPA administration in AIS patients.

In 2004 a pooled analysis of the data from NINDS, ECASS, and ATLANTIS trials suggested two interesting conclusions: [1] a greater benefit was observed when tPA administered to AIS patients within 90 min of stroke onset, upholding the concept of “sooner the better” and [2] a potential benefit beyond 3 h of tPA administration was also observed, however with some risks (23). This second conclusion prompted the initiation of ECASS III study, at the request from European regulators, to confirm the benefit of the tPA administration beyond 3 h (3). After assessing data from 821 patients, the results of the study demonstrated a significantly improved clinical outcome in AIS patients when tPA was administered between 3 and 4.5 h of stroke onset. A meta-analysis of these major trials also supported the benefit of tPA when administered between 0 and 4.5 h of stroke onset in AIS patients (24).

In most of the above studies, elderly patients, aged >80 years, were under-represented (25, 26), which resulted in regulatory restriction of tPA use in this group of AIS patients. This was addressed in a decade long study, “Third International Stroke Trial” (IST-3), the results of which revealed that the >80 year old AIS patients were equally benefited from tPA treatment as in younger patients (27). Further the study assessed a therapeutic window of 6 h of stroke onset for tPA administration and claimed an improved functional outcome in the recruited patients, despite early hazards. A comprehensive individual patient meta-analysis of data from 6756 AIS patients enrolled in all the above clinical trials was carried out to assess the effect of therapeutic window, age and stroke severity on the outcome of tPA therapy (28). The results confirmed a generalized efficacy of tPA across age and stroke severity spectra with a good functional outcome when delivered within 4.5 h of stroke onset, although an increased risk of fatal ICH was evident in the early days of treatment.

**Figure 4:**
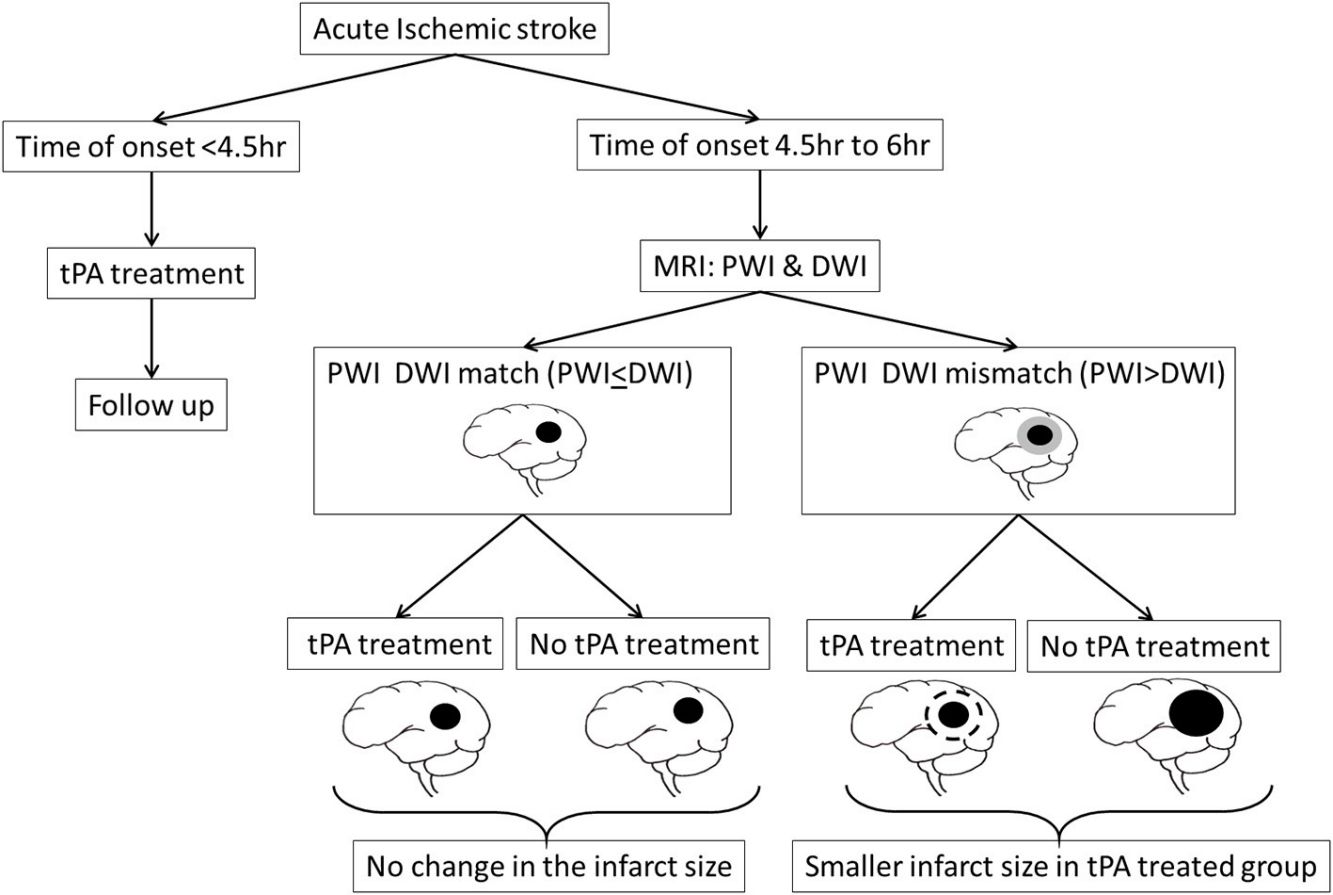
MRI could identify potentially salvageable brain tissue, as indicated by a mismatch between Diffusion-weighted imaging (DWI) and Perfusion-weighted imaging (PWI), in a subset of AIS patients who might then benefit from tPA therapy beyond its 4.5 h therapeutic window.

These studies relied on traditional CT scan to image early infarct signs in AIS patients and on Alberta stroke program early CT (ASPECT) score to decide the risk of hemorrhagic transformation. However, by 1999 considerable number of studies were indicating MRI techniques, particularly the mismatch between Diffusion-weighted imaging (DWI) and Perfusion-weighted imaging (PWI), could identify potentially salvageable brain tissue in a subset of AIS patients who might then benefit from tPA therapy beyond its 3 h window (Figure 4) (29). This triggered a small phase-2 randomized placebo controlled trial, referred to as “echoplanar imaging thrombolytic evaluation trial” (EPITHET), which targeted AIS patients between 3 and 6 h post stroke onset (30). The results indicated that in mismatch patients, tPA could attenuate infarct growth and increase reperfusion, which translated into improved clinical outcomes even when tPA was administered beyond its 3 h window (30, 31). When imaged immediately before and 3–6 h post tPA treatment, MRI was also successful in identifying AIS patients who are likely to clinically respond to early reperfusion treatments (32).

EXTEND and ECASS-4:EXTEND are two other studies which assessed the effectiveness of perfusion imaging guided tPA thrombolysis at up to 9 h of AIS onset, with contradicting results (33–36). The EXTEND study found excellent functional outcome in perfusion mismatch AIS patients, compared to placebo group, when tPA was administered between 4.5 and 9 h of stroke onset. However, the ECASS-4:EXTEND failed to elicit any significant benefits of alteplase administered at the same therapeutic window. Considering the modest sample size in each of these trials, a meta-analysis was carried out involving data from EXTEND, ECASS-4:EXTEND and EPITHET trails the results of which implicate that the AIS patients with perfusion mismatch, suggesting of salvageable brain tissue, do demonstrate improved functional outcome even when the tPA was administered beyond its 4.5 h window, for up to 9 h of stoke onset (37). Few patients reach the emergency department with unknown time of stroke onset, making them ineligible for tPA therapy on timing alone. MR WITNESS and WAKE-UP are two randomized trials which addressed this issue with the help of MRI-guided thrombolysis (38, 39). Both MR WITNESS and WAKE-UP studies concluded that it is safe and effective to administer tPA guided by the diffusion mismatch and perfusion mismatch in AIS patients, respectively, with unknown time of onset, which compels the importance of “tissue window” and not “time window” in AIS management with tPA (40). Based on these results, the 2019 guidelines from American Heart Association/American Stroke Association (AHA/ASA) gave a class IIa recommendation to consider the AIS patients with unclear time of onset or who wake-up with stroke symptoms, in whom MRI show a mismatch between DWI and FLAIR signals, no signs of intracranial hemorrhage or the lesions not larger than 1/3rd of the territory of the middle cerebral artery, and the NIHSS score of <25, for tPA administration within 4.5 h from the stroke symptom recognition (41). In 2015 five randomized clinical trials (MR CLEAN, ESCAPE, EXTEND IA, SWIFT PRIME, and REVASCAT) were published reporting the efficacy of endovascular thrombectomy in AIS patients with proximal arterial occlusion (42–46). A meta-analysis of these trials revealed that by bridging tPA with intrarticular thrombectomy the treatment window could be expanded up to 6 h (4). In clinical settings, when eligible, intravenous thrombectomy is often combined with endovascular thrombectomy in a process referred to as bridging-therapy. The efficacy of bridging-therapy over endovascular therapy alone is controversial with few studies showing no added benefit (47–50), while other studies demonstrating considerable benefits (51–53). Of interest, a recent study reported that bridging-therapy resulted in improved cognitive function in patients with large cerebral vessel occlusion stroke, at 90 days after index ischemic stroke, compared to endovascular therapy alone (54). This demonstrates that the intravenous tPA administration has an added advantage when combined with mechanical thrombectomy and administered within the specified timeline.

Initially, the NINDS stroke trial group conducted a workshop which concluded to propose a global statistic to test the trial's primary hypothesis and secondary tests of individual outcomes, and familiarize the clinical/scientific community with such global approach (55). However, it was not well-received by the community leading to a considerable heterogeneity in the outcome measures across the stroke trials since then (56). Such heterogeneity can be attributed to the availability of several assessment tools, which focuses primarily on either measuring neurological impairment or on the degree of functional independence of the patient. Further, difference in timing of the assessments and data collection in stroke trials also contributes to the observed heterogeneity, all of which makes it extremely difficult to compare between the studies and draw meaningful conclusions from the large number of published stroke trials.

## tPA in Current Clinical Settings: How The Past Has Shaped The Present

Following NINDS trial results USFDA approved alteplase for treating AIS patients within 3 h of stroke onset. The drug label portray inclusion and exclusion criteria of NINDS trial, with the following contraindications: current intracranial hemorrhage, subarachnoid hemorrhage, active internal bleeding, intracranial/intraspinal surgery or serious head trauma in the past 3 months, intracranial conditions that may increase risk of bleeding, bleeding diathesis, and current severe uncontrolled hypertension. In Europe, alteplase was approved with more stringent exclusion criteria which also excluded AIS patients with: >80 years of age, on anticoagulant therapy, history of stroke and diabetes, and current severe stroke. Later, following ECASS III study results, European Union relaxed the inclusion and exclusion criteria to mirror that of the ECASS III criteria, and the treatment window was extended to 4.5 h. The age and stroke severity was also relaxed based on the results of the meta-analysis of data from 6,756 patients enrolled in major trials (28). The results of the meta-analysis reveal that, despite higher rate of ICH and mortality as compared to <80 years old patients, >80 years old patients treated with tPA within 3 h of onset of stroke symptoms demonstrate a better chance of being independent at 3 months. Further, the analysis also establish a clinical benefit of tPA therapy in AIS patients with severe stroke symptoms and in patients with mild but disabling stroke symptoms, both of which were considered as exclusion criteria previously. This treatment label holds good in most of the countries across the world, except USA and Canada, who follow 3 h window. However, AHA/ASA and American Academy of Neurology have endorsed this extended time window (57). Further, AHA/ASA has issued statement that additional criteria from ECASS III should not be considered as strict exclusion criteria for tPA therapy in AIS patients (58). Considering the results from perfusion imaging mismatch studies the current AHA/ASA guidelines (2019) has given a class IIa (moderate) recommendation to select patients who wake up with symptoms for alteplase treatment (41). Influenced by DAWN study results, mechanical thrombectomy up to 24 h of stroke onset has also been given a status of class 1A recommendation by AHA/ASA in its 2019 guidelines. The FDA prescribing information (PI) for alteplase was also updated (58) https://www.accessdata.fda.gov/drugsatfda_docs/label/2015/103172s5203lbl.pdf), which now extended the age of the AIS patients to 77 years after which a warning was added. Further, the contraindications like prior stroke within 3 months and seizure at onset are now removed completely from the PI. Further, the warnings on administering tPA to AIS patients with low blood glucose (<50 mg/dl) level, NIHSS>22 or mild stroke are also lifted. History of ICH, which was a contraindication for tPA use in AIS patients initially has been removed now with a warning added for recent ICH. However, current ICH, subarachnoid hemorrhage and severe uncontrolled hypertension remains contraindicated. In this way, along with the tPA therapeutic window, the inclusion criteria of AIS patients for tPA therapy has also evolved over time.

## Complications of Alteplase Therapy and Its Management

Alteplase administration in AIS patients can induce symptomatic/fatal intracerebral hemorrhage, systemic hemorrhage, and angioedema in about 6, 2, and 5% of the patients, respectively (59). The risk of hemorrhage increases with age and stroke severity with large areas of ischemic change and leukoaraiosis. Associated conditions like obesity, hyperglycemia, hypertension, cardiac conditions, and use of antiplatelet agents also increases the risk of hemorrhage. A gender bias, with male patients demonstrating increased risk of hemorrhage, is also reported. In addition to regulating the underlying condition, it is recommended to manage hemorrhage with cryoprecipitate or protein complex concentrate, although literature on its benefits are feeble (60). Patients on angiotensin-converting enzyme (ACE) inhibitor are at a higher risk for angioedema, which when involves oropharynx can compromise airway. It is essential to act quickly, typically managed with steroids, antihistamines and intubation if needed. AHA/ASA guidelines recommend the stroke centers to have systems (protocols, personnel, and devices) to manage such complications (41).

## Emerging Strategies and Future Directions of Evolution

The evolution of tPA therapy is directed toward ways to increase the number of AIS patients eligible for the therapy either by speeding up the patient recruitment, so that they fall within the recommended therapeutic window, or by expanding the window with added therapies.

### Speeding Up Patient-Recruitment for tPA Therapy

Current guidelines recommend to transport AIS patient directly to comprehensive stroke center when the travel time is <15 min, than to other hospitals (61). However, this is not always possible, especially in patients living in non-urban areas. To address the issue, telemedicine, a growing field of technology, is being adopted in stroke management programs. Referred to as telestroke, it facilitates audiovisual communication where neurologists from a stroke center can guide the physicians at a place with limited access to neurologists, thus enabling a safer and quick management of AIS patients. Studies have determined the technology as safe and effective (62) and thus bags IIa recommendation from AHA/ASA, as the clinical outcomes over long run is not clear yet (63). Considering the importance of time window, few centers implemented mobile stroke units to minimize the tPA window (64). However, this is an expensive venture requiring a fully functional CT scan machine within the ambulance along with a Neurologist, critical care nurse, and CT technician, which makes it impractical at many centers across the globe. Further, the data on the outcome of AIS patients availing the advantage of mobile stroke unit, vs. those not, is limited to conclude the advantage of such system. A delay in door-to-needle time has been associated with poor outcome in AIS patients (65), especially in posterior circulation stroke (PCS) conditions (66). Thus, efforts are made to decrease the door-to-needle time, although the benefits of such efforts are yet to be established (67, 68).

An efficient organization of a stroke care system is equally important to ensure short treatment time, toward which several recommendations and guidelines have been established. A recent update on policy statement from American Stroke Association (ASA) has listed the essential framework of the stroke care system composed of eight domains as follows: community education, primordial prevention, primary prevention, emergency medical service (EMS) response, acute stroke treatment, secondary stroke prevention, stroke rehabilitation and continuous quality improvement (61). The first three domains work as preventive strategies. Educating the community is also extremely helpful in quickly identifying a stroke victim and use the EMS, the 4th domain, to reach the correct hospital in time, eventually contributing in reducing patient's time-to-treatment. EMS providers should be educated and trained well to recognize the stroke symptoms, reduce the on-scene time, prenotify the nearest stroke center, and transport without delay, to make sure that the victim receives the treatment as soon as possible. Certifying the hospitals will be of help to identify the correct hospital, toward which USA has designated 4 levels of hospital certification as follows: comprehensive stroke center, primary stroke center, acute stroke-ready center, and thrombectomy-capable stroke center. Once patient arrives at the hospital, the hospital should have parallel processes running to keep the door-to-treatment time as short as possible, toward which the stroke centers are modifying and implementing new patient centered protocols achieving better door-to-needle time (67–69). Establishing mobile stroke units and telemedicine contributes to completing patient assessment, at least in part, before the arrival of patient at the hospital, eventually reducing the door-to-needle time. The last three domains are important in preventing the recurrence of the condition and improving the quality of life of the stroke patients, toward which new models of care with patient-centered-approach are being implemented by the stroke centers.

NIHSS is the most widely used scale to assess the AIS patients. However, Burns JD and team reported a significant variation in scoring time between stroke variants when using NIHSS, where it took 504 min to score basilar artery occlusion as compared to 83 min for left middle cerebral artery occlusion (70). This indicates that NIHSS is heavily weighted toward ACS symptoms and not PCS symptoms (66, 71), which may explain the increased door-to-needle time noted specifically in PCS conditions in results from Australian stroke unit registry (66), resulting in extended NIHSS scale (72). Recently, NIHSS was reported as not enough to detect cognitive deficits in AIS, demanding the need for improvisation of the concerned section (73). As mentioned above, availability of various assessment scales adds to the heterogeneity in the outcome measures across the stroke trials, demanding the need for a more comprehensive scale that is acceptable across the clinical/scientific community. These conditions suggests a clear need for an improved stroke scoring scale that is both accurate and quick, aiding in accelerated recruitment of right AIS patient for tPA therapy.

### Expand the Window for tPA Therapy

Imaging has significantly expanded the time window of a certain subgroup of AIS patients, the details of which are discussed above. However, although studies have showed the benefit of thrombolysis guided by perfusion imaging in AIS patients up to 9 h of stroke onset (37), the current guidelines has not recommended it, yet, indicating the need for more proof on the concept. Also, image-guided tPA administration in wake-up AIS patients, for up to 4.5 h from stroke symptom recognition, has received only moderate recommendation (class IIa), indicating conflicting evidence and/or a divergence of opinion about its efficacy, thus demanding more work in the area. The DWI shows us the ischemic core, which is considered irreversible part of the lesion. However, post treatment (tPA or endovascular thrombectomy) follow-up imaging from few early studies indicates that some of these lesions are reversible if blood flow is restored promptly (74–76), although there is uncertainty about this phenomena. A recent systematic review, assessing the results of such studies, confirmed the existence of such reversible DWI lesions and associated them with good clinical outcome following reperfusion (77). The study also highlights the pitfalls of DWI in defining such lesions early enough, opening the window for improvements in the area which might help in redefining ischemic core in early hours of stroke. Thus, imaging the stroke lesion and its surrounding blood vessels, including pial collateral vessels that significantly preserve the brain tissue in the penumbral region (78, 79), using modern imaging techniques is a hot topic of research in stroke management.

Increase in risk-to-benefit ratio over time, limits the tPA therapeutic window in AIS patients. The most common and devastating risk is HT, addressing which can not only expand the tPA treatment window but also can improve its safety profile. Pre-clinical studies have explored several pharmacological and non-pharmacological agents that, when administered with tPA, can stabilize BBB, preserve neurovascular function, attenuate inflammation and offer neuroprotection (80, 81), possibly extending its therapeutic window. Several of such agents have undergone human studies with intriguing, yet unsatisfactory, results, the details of which are discussed in recent narratives (71, 82). Clinically, a subpopulation of AIS patients who are resistant to tPA has been identified, who fail to respond to tPA treatment even when administered within the approved time-window (83, 84). The factors underlying this phenomena is not well-understood, although thrombus location, its size and composition, and the age of the thrombus are known to play a crucial role (85, 86). Biologically two types of thrombus can be identified; erythrocyte-rich thrombi and platelet-rich thrombi, and their resistance to tPA thrombolysis has been variedly reported (87–89). In this regards, few pharmacological agents that target other components of the thrombi, von willibard factor (vWF) meshwork or neutrophil extracellular traps (NETs), show promise to overcome tPA resistance (85). During thrombosis, vWF multimers are involved in crosslinking platelets to fibrin, and vWF lysis can thus be promising in platelet-rich thrombi. In experimental stroke models, N-Acetylcysteine, a mucolytic drug, and ADAMTS13, a vWF cleaving enzyme, have shown to effectively dissolve tPA resistant thrombi and reduce cerebral infarct size (90, 91). During thrombosis, neutrophils stabilize the thrombus structure by releasing DNA filaments composed of histones and cytoplasmic granule proteins, referred to as NETs, which are shown to impair tPA-induced thrombolysis (92). Deoxy-ribonuclease (DNAse) enzyme can counteract the NETs activity and has shown protective effect in experimental stroke model (93). Further, under *in vitro* condition, DNAse has shown to augment the tPA mediated thrombolysis and thus is of clinical interest (92, 94). Clearly much needs to be achieved before clinical translation of these additive therapies, which when done are promising to add a significant time to the current tPA therapeutic window.

### Modified Tissue Plasminogen Activators

The limitations of alteplase, such as increased bleeding risk, potential neurotoxicity, and short half-life (about 5 min) (8, 11–13, 95), have lead to the development of new thrombolytic agents over the past 25 years, that includes reteplase, tenecteplase, desmoteplase, monteplase, pamiteplase, lanoteplase, and alfimeprase (96, 97). Of these, tenecteplase and desmoteplase demonstrate longer plasma half-life, improved fibrin specificity, better resistance to plasminogen activator inhibitor and lower neurotoxicity, and show promise in treating AIS (98). At 3–6 h of stroke onset, 0.1 mg/kg of tenecteplase was superior to 0.9 mg/kg of alteplase in achieving better reperfusion, recancanalizaion with improved NIHSS score in AIS patients at 24 h (99). Importantly, tenecteplase allowed the expansion of the treatment window to 6 h with better efficacy than alteplase at both 24 h and 90 days, as confirmed in phase 2 and phase 3 trials (100, 101). Based on the results of these studies, current clinical guidelines suggest intravenous tenecteplase, at a dose of 0.25 or 0.4 mg/kg, as a second-tier option in treating large vessel occlusions (101). Desmoteplase is a recombinant version of DSPAα1, a plasminogen activator isolated from the saliva of the vampire bat (102). Due to its high fibrin specificity and low neurotoxicity, desmoteplase is a promising thrombolytic agent currently under trials for treating AIS. Meta-analsysis of these clinical trials conclude that desmoteplase is associated with a favorable reperfusion and acceptable safety when administered after 3 h and up to 9 h of onset of stroke symptoms (103, 104). More trials are underway to assess its true efficacy in AIS patients.

## Conclusion

Time is the mantra of brain, and is of prime consideration when it comes to managing stroke. Any patient with AIS presenting to hospital within 4.5 h of symptom onset should be promptly treated with intravenous tPA, unless otherwise contraindicated. To truly maximize the benefit of tPA therapy, one should reduce the onset of tPA administration to the earliest possible time. Studies have demonstrated that every minute saved in achieving reperfusion translates into 1.8 days (mean) of “disability adjusted life years” in AIS patients, while loss of every 20 min increased the “number needed to treat” to achieve excellent outcome by 1 (95). Further, a significant reduction in hospital stay, in-hospital mortality and other adverse effects of tPA administration was reported with a 15 min reduction in door-to-needle time (105). Systems/protocols to minimize the door-to-needle time to the shortest possible period must be implemented in all stroke centers, without compromising safety. It is recommended to train critical care physicians on recognition of stroke symptoms and stroke screening tools which may reduce the door-to-needle time (106). It is believed that the number of AIS patients receiving tPA therapy remains much lower than what is achievable. In this regard, it is recommended to educate public on early detection of stroke symptoms that may facilitate early transportation of such stroke patients to critical care centers. Systems like mobile stroke unit and telestroke services hold great promise to include more AIS patients under the tPA umbrella. “Tissue window” should be considered when a patient reaches beyond the recommended 4.5 time window, which may qualify many perfusion mismatch patients to tPA therapy. Improving the safety profile of tPA therapy with combination therapies are further warranted, which may also contribute to improved clinical outcomes. Further, such therapies may also extend the current tPA therapeutic window, qualifying many more AIS patients for tPA therapy who are otherwise deemed ineligible currently.

## Author Contributions

YP and GS: substantial contributions to the conception, design of the work and acquisition of data for the work, drafting the work or revising it critically for important intellectual content, final approval of the version to be published and agreement to be accountable for all aspects of the work in ensuring that questions related to the accuracy or integrity of any part of the work are appropriately investigated and resolved.

## Conflict of Interest

The authors declare that the research was conducted in the absence of any commercial or financial relationships that could be construed as a potential conflict of interest.
